# 652 Scald Burns in Children Under 3 Years: Updates on Prevention Efforts Using the NEISS Database

**DOI:** 10.1093/jbcr/iraf019.281

**Published:** 2025-04-01

**Authors:** Mary Froehlich, John Schomberg, Theresa Chin, Laura Goodman

**Affiliations:** Kirk Kerkorian School of Medicine at UNLV; Children’s Hospital of Orange County; University of California Irvine; Children’s Hospital of Orange County

## Abstract

**Introduction:**

Thermal injury is a leading cause of unintentional trauma in pediatrics. This is especially true for children in the youngest age group who are susceptible to deeper burns given their thin skin and subcutaneous tissue. Notably, scald burns account for a significant portion, constituting 50% of all pediatric burn injuries. The aim of this study is to identify trends and patterns in the epidemiology of scald injuries in pediatric patients using a national database.

**Methods:**

The National Electronic Injury Surveillance System (NEISS) was queried for all cases of scald injury in patients up to 3 years old between 2013 and 2022. The data set was then reviewed to identify injury patterns.

**Results:**

The NEISS query identified 143820 cases between the ages of 0 and 3 years, which resulted in a national estimate of 125133 scald burns. Most (81%) of the burns occurred at home (n=116394). Stratified by anatomic region, 35% of the children sustained burns on their trunk (n=50286). Among all children, the incidence of burns from appliances decreased over time. Those related to grabbing or pulling non-appliances increased by 80.7% (2889 to 5223). The proportion of injuries from bathing was greater in patients ages 1-5 months who required transfer or admission (1814, 51.4%) compared to those who were treated and released (1940, 30.3%, p< 0.0001). Furthermore, children age 0-6 months experienced an increase in the incidence of burn injury over the last decade (1389 to 2381).

**Conclusions:**

Burn education should focus on teaching safe bathing techniques to prevent severe burns, especially in infants. Additional educational campaigns should focus on safe placement of hot liquids to prevent pulling/grabbing related burn injuries.

**Applicability of Research to Practice:**

This study on pediatric scald injuries highlights two key prevention areas: safe bathing practices and preventing burns from children pulling or grabbing hot objects. Most burns occur at home, with infants being particularly vulnerable, especially during bathing. Education on maintaining safe water temperatures and keeping hot liquids out of children’s reach is essential for reducing these injuries. The research shows an increase in scald burns over the last decade, emphasizing the need for targeted prevention efforts. By applying these findings, healthcare providers can help lower the incidence of severe burns in young children through focused educational campaigns.

**Funding for the Study:**

N/A

